# Conventional glaucoma implants and the new MIGS devices: a comprehensive review of current options and future directions

**DOI:** 10.1038/s41433-021-01595-x

**Published:** 2021-06-14

**Authors:** Inês C. F. Pereira, Rosanne van de Wijdeven, Hans M. Wyss, Henny J. M. Beckers, Jaap M. J. den Toonder

**Affiliations:** 1 Microsystems Research Section, Department of Mechanical Engineering, Eindhoven University of Technology, Eindhoven, The Netherlands; 2 Institute for Complex Molecular Systems (ICMS), Eindhoven University of Technology, Eindhoven, The Netherlands; 3 University Eye Clinic Maastricht, Maastricht University Medical Centre+ (MUMC+), Maastricht, The Netherlands

**Keywords:** Optic nerve diseases, Surgery, Implants

## Abstract

Glaucoma is a progressive optic neuropathy that is the second leading cause of preventable blindness worldwide, after cataract formation. A rise in the intraocular pressure (IOP) is considered to be a major risk factor for glaucoma and is associated with an abnormal increase of resistance to aqueous humour outflow from the anterior chamber. Glaucoma drainage devices have been developed to provide an alternative pathway through which aqueous humour can effectively exit the anterior chamber, thereby reducing IOP. These devices include the traditional aqueous shunts with tube-plate design, as well as more recent implants, such as the trabeculectomy-modifying EX-PRESS^®^ implant and the new minimally invasive glaucoma surgery (MIGS) devices. In this review, we will describe each implant in detail, focusing on their efficacy in reducing IOP and safety profile. Additionally, a critical and evidence-based comparison between these implants will be provided. Finally, we will propose potential developments that may help to improve the performance of current devices.

## 摘要

青光眼是一种进行性视神经病变, 为全球仅次于白内障的第二大可预防性致盲性眼病。眼压 (IOP) 升高被认为是青光眼的主要危险因素, 并与前房房水流出阻力的异常增加有关。青光眼引流装置为房水有效地排出前房提供了一种新的途径, 从而降低眼压。这些装置包括传统的具有管板设计的房水分流器, 以及最新的植入物, 如小梁切除术改良EX-PRESS®植入物和新的微创青光眼手术 (MIGS) 装置。在这篇综述中, 我们将详细描述每种植入物, 并重点介绍它们在降低眼压和安全性方面的功效。此外, 本文对这些植入物进行关键的循证的比较。最后, 我们将提出有助于改善现有装置的性能进行潜在改善的建议。

## Introduction

**Fig. 1:**
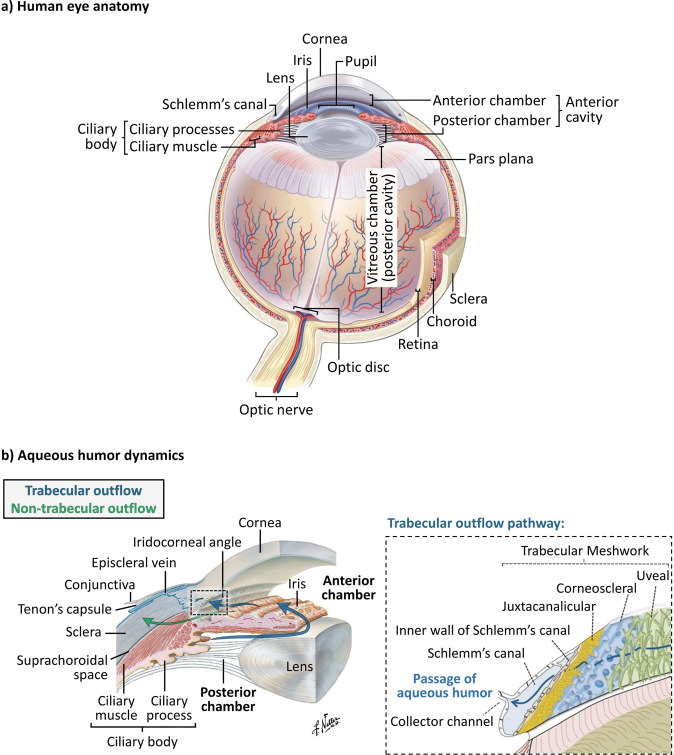
Anatomy of the human eye and aqueous humour dynamics. **a** Schematic representation of the anatomy of the human eye [9]. **b** Schematic representation of the aqueous humour dynamics inside the anterior cavity of the eye, where the blue arrows represent both the production/secretion of aqueous humour and its drainage via the trabecular outflow pathway, whereas the green arrow represents the non-trabecular outflow pathway; the anatomical structures involved in the trabecular outflow pathway, including the trabecular meshwork, Schlemm’s canal and collector channel, are represented in the figure on the right; images reproduced with permission from [8] and [10].

Glaucoma is a progressive optic neuropathy characterised by optic nerve damage and visual field loss [1]. It is the leading cause of irreversible blindness in the world, with over 70 million people affected and 10% being bilaterally blind [2]. Patients suffering from this disease are asymptomatic until later stages, when significant and irreversible visual impairment has already taken place [3, 4]. Elevated intraocular pressure (IOP, above 21 mmHg) is the most important known risk factor for the development and progression of patients with ocular hypertension and primary open-angle glaucoma. It results from an unbalance between production and drainage of aqueous humour, the fluid that circulates inside the anterior and posterior chambers of the eye [5, 6]. Aqueous humour is produced by the ciliary processes within the posterior chamber, and then flows anteriorly around the lens and through the pupil, filling the anterior chamber (see Fig. 1a, b [7–10]). From there, aqueous humour drains at the iridocorneal angle via two routes: the trabecular and the non-trabecular pathways [11]. The trabecular outflow pathway is considered to be the major site of aqueous humour outflow, and is anatomically comprised of the trabecular meshwork (subdivided into uveal, corneoscleral, and juxtacanalicular meshworks), Schlemm’s canal, collector channels, and the episcleral veins, as represented in Fig. 1b [8, 11]. Within this pathway, the juxtacanalicular meshwork and the inner wall of Schlemm’s canal have been shown to be the key source of outflow resistance that leads to increased IOP [12, 13]. A minor fraction of the aqueous humour also drains through an alternative route, the non-trabecular outflow pathway. Here, the aqueous humour flows through the ciliary muscle into the suprachoroidal space (indicated in Fig. 1b) [11, 14–16].

Current treatment options for glaucoma are focused on lowering IOP, which remains the only proven treatment for stopping vision loss progression up to now. This can be achieved by different methods, including pharmacological medication, laser treatment, and surgery [17, 18]. Surgical intervention is required when there is progressive optic neuropathy as indicated by worsening disc/retinal nerve fibre layer parameters and/or visual fields changes, or indeed in the case of very high IOPs without significant disc damage, despite prior pharmacological, and/or laser treatment [19]. Conventional filtration surgeries include trabeculectomy and implantation of glaucoma drainage devices, also known as aqueous shunts. Both surgical procedures are based on the same principle: bypassing the eye’s natural outflow pathways to provide an alternative route for aqueous humour to effectively exit the anterior chamber, thereby reducing IOP [18, 20]. In trabeculectomy, a fistula is created into the anterior chamber from underneath a scleral flap (ab externo approach), which allows the aqueous humour to drain from the anterior chamber into the sub-Tenon’s space (space formed between the Tenon’s capsule and sclera, see Fig. 1b), forming a subconjunctival reservoir of aqueous humour referred to as filtering bleb [21]. Conventional aqueous shunts drain aqueous humour via a tube inserted into the anterior chamber to a sub-Tenon’s end plate, creating a more posteriorly located bleb [20]. While these devices were traditionally reserved for high-risk patients or after trabeculectomy had failed, they are increasingly used as a primary procedure [2, 22]. However, despite being efficacious at lowering IOP, these incisional surgeries are associated with possible serious postsurgical complications and require substantial postoperative management. Thus, in order to provide a safer and less invasive method of reducing IOP, a new class of glaucoma drainage devices and procedures has recently emerged, termed minimally- or micro-invasive glaucoma surgery (MIGS) [18, 23, 24]. Regardless of the procedure or device used, the overall goal of surgical treatment is to reduce IOP to a level that will prevent further damage of the optic nerve, typically around 10 mmHg [25].

This review will focus on several of the currently available glaucoma drainage devices, including conventional aqueous shunts (tube-plate design) and more recent implants, such as the EX-PRESS^®^ Glaucoma Filtration Device and the new MIGS devices. We will describe each implant in detail, highlighting their efficacy in reducing IOP and their safety profile (see Table 1 [26–36]). A critical and evidence-based comparison of the devices will then be provided. Finally, we will provide our opinion about the future directions of this growing field.

**Table 1:** Conventional glaucoma drainage devices, the trabeculectomy-modifying EX-PRESS^®^ device, and the new MIGS devices—summary of the current regulatory status, advantages, and disadvantages of each group of devices (aqueous shunts—valved and non-valved; MIGS devices—Schlemm’s canal, suprachoroidal, and subconjunctival devices), and effectiveness of each device in reducing IOP, as reported in selected studies.

Type of device	Site of anatomical intervention	Device (Manufacturer)	Regulatory status	Advantages	Disadvantages	% IOP reduction at follow-up (Study type)
Conventional glaucoma drainage devices	Subconjunctival space	Molteno^®^ Glaucoma Drainage Device (Molteno Ophthalmic Limited)	CE mark, FDA approval	Larger surface area of end plate provides greater long-term IOP reduction	Delayed functioning until encapsulation of plate occurs (high IOP in the postoperative period); Greater risk of postoperative hypotony and hypotony-related complications	53.4% at 2 years (Prospective, randomised clinical trial) [26]
		Baerveldt^®^ Glaucoma Implant (Johnson & Johnson Vision)	CE mark, FDA approval			57.4% at 5 years (Prospective, randomised clinical trial) [27]
		PAUL^®^ Glaucoma Implant (Advanced Ophthalmic Innovations)	CE mark granted in 2017	Smaller tube diameter occupies less space in the anterior chamber angle	Complications reported so far: shallow anterior chamber, tube occlusion and exposure, hypotony requiring intervention and endophthalmitis	42.9% at 1 year (Prospective, single-arm clinical trial) [38]
		Ahmed^®^ Glaucoma Valve (New World Medical, Inc.)	CE mark, FDA approval	Valve minimises risk of postoperative hypotony and hypotony-related complications; Allows immediate IOP reduction	Higher rate of bleb encapsulation and smaller surface area of end plate may decrease IOP-reducing efficacy; Valve malfunction can result in hypotony and hypotony-related complications	46.6% at 5 years (Prospective, randomised clinical trial) [27]
		Ahmed^®^ ClearPath Glaucoma Drainage Device (New World Medical, Inc.)	CE mark, FDA approved since 2019	Equivalent to Baerveldt implant	Equivalent to Baerveldt implant	Equivalent to Baerveldt implant
Trabeculectomy- modifying device	Subconjunctival space	EX-PRESS^®^ Glaucoma Filtration Device (Alcon Laboratories, Inc.)	CE mark, FDA approved since 2002	High efficacy in reducing IOP; More predictable than trabeculectomy, with less IOP fluctuations during the early postoperative period; Complications are less frequent when compared to trabeculectomy	Risk of failure as a consequence of subconjunctival fibrosis and bleb-related complications; hypotony is commonly reported, as well as erosion, displacement, and blockage of the implant	41.4% at 2 years (Prospective, randomised clinical trial) [29]
Minimally invasive glaucoma surgery (MIGS) devices	Schlemm’s canal	iStent^®^ (Glaukos Corporation)	CE mark granted in 2004, FDA approved since 2012	Lower risk of hypotony and hypotony-related complications; Favourable safety profile	High risk of fibrosis that may lead to device obstruction; Modest IOP reduction, which makes these devices only suitable for patients with mild-to-moderate glaucoma; Not suitable for patients with high episcleral venous pressure	33.7% at 1 year (Prospective, randomised clinical trial) [30]
		iStent inject^®^ (Glaukos Corporation)	CE mark granted in 2010			31.0% at 2 years^a^ (Prospective, randomised clinical trial) [31]
		iStent inject^®^ W (Glaukos Corporation)	FDA approval since 2020			No published studies
		Hydrus^®^ Microstent (Ivantis, Inc.)	CE mark granted in 2011, FDA approved since 2018			37.1% at 1 year (Prospective, randomised clinical trial) [30]
	Suprachoroidal space	CyPass^®^ Micro-Stent (Alcon Laboratories, Inc.)	Withdrawn from the global market in 2018	More effective at reducing IOP as compared with Schlemm’s canal MIGS devices	High risk of fibrosis that may lead to device obstruction; Unpredictable IOP spikes; Higher risk for (transient) hypotony	34.3% at 5 years^a^ (Prospective, randomised clinical trial) [32]
		iStent SUPRA^®^ (Glaukos Corporation)	CE mark granted in 2010, under FDA review			No published studies
		SOLX^®^ gold shunt (SOLX, Inc.)	CE mark granted, approved in Canada			35.8 % at 5 years (Prospective, randomised clinical trial) [33]
		STARflo™ Glaucoma Implant (iSTAR Medical)	CE mark granted in 2012			38.5% at 2 years (Prospective study) [34]
		MINIject™ (iSTAR Medical)	Application for CE marking expected in 2020			39.1% at 6 months (Prospective, single-arm clinical trial) [35]
	Subconjunctival space	XEN® Gel Stent (Allergan, Inc.)	CE mark granted in 2013, FDA approval since 2016	High efficacy in reducing IOP, making these devices suitable for patients with more severe glaucoma; Possibility of applying antifibrotic agents in the subconjunctival space optimises the fibrotic response; Modulating bleb encapsulation is possible with techniques such as bleb massage or bleb needling	Risk of failure as a consequence of subconjunctival fibrosis and bleb-related complications	36.3% at 1 year (Prospective, single-arm clinical trial) [36]
		PRESERFLO™ MicroShunt (Santen)	CE mark granted in 2012, under FDA review			46.7% at 5 years (Prospective, nonrandomized, single-arm clinical trial) [101]

^a^In combination with cataract surgery.

### Conventional glaucoma drainage devices

Conventional glaucoma implants fall into two categories: valved or non-valved devices, depending on whether a valve mechanism is present to help prevent hypotony, usually in the early postoperative phase [25]. Hypotony is defined as an IOP of 5 mmHg or less, and it may lead to vision loss in up to 20% of patients. It can be accompanied by a shallow anterior chamber, hyphema (collection of blood inside the anterior chamber), but it may also lead to more devastating complications (e.g. choroidal effusions/haemorrhage) [37].

**Fig. 2:**
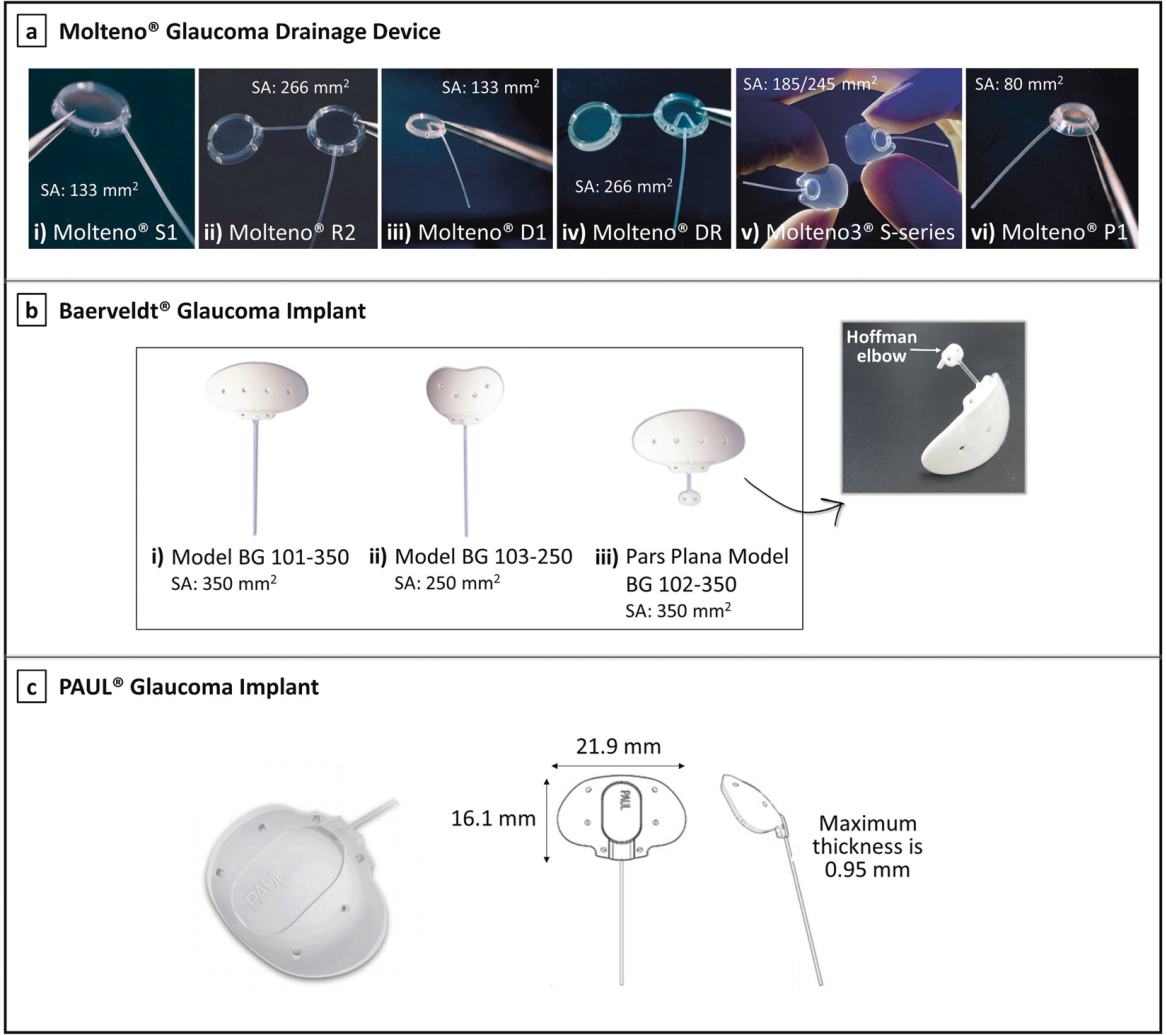
Molteno, Baerveldt, and PAUL implants. **a** The Molteno® implants: (i) Molteno^®^ single plate implant S1, the original Molteno^®^ glaucoma implant; (ii) Molteno^®^ double plate implant, available in right eye (R2) and left eye (L2) configurations; (iii) Molteno^®^ pressure ridge single plate implant D1; (iv) Molteno^®^ pressure ridge double plate implant, available in right eye (DR) and left eye (DL) configurations; (v) Molteno3^®^ S-series, with the end plate available in two different sizes: 185 mm^2^ (SS, left side) and 245 mm^2^ (SL, right side); and (vi) Molteno^®^ microphthalmic implant P1 [39]; images courtesy of Molteno Ophthalmic Ltd. **b** The Baerveldt^®^ implants: (i) Baerveldt^®^ BG 101–350; (ii) Baerveldt^®^ BG 103–250; and (iii) Baerveldt^®^ Pars Plana BG 102–350, showing its Hoffman elbow that allows positioning the tube into the vitreous cavity; images reproduced with permission from [25] and [135]. **c** The PAUL^®^ Glaucoma Implant, showing the dimensions of the end plate [38, 136]; left image courtesy of Advanced Ophthalmic Innovations, and right image reproduced with permission from [38]. “SA” stands for surface area of the end plate.

**Fig. 3:**
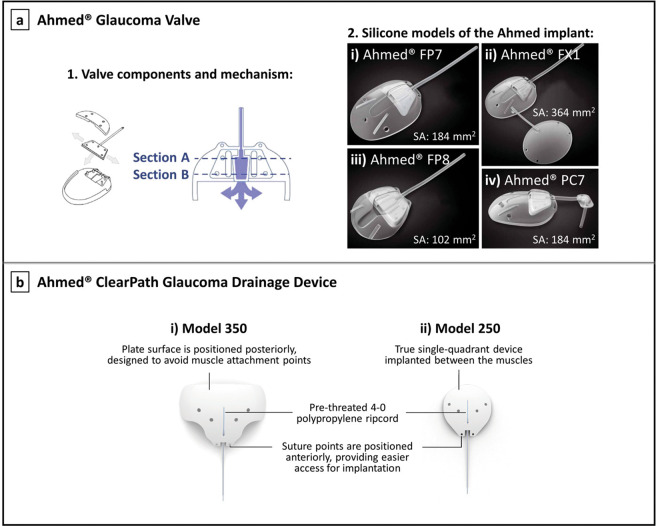
Ahmed implants. **a**-1 The Ahmed^®^ Glaucoma Valve showing its components and valve mechanism, where Section A represents the larger inlet port of the integrated Venturi chamber, and Section B represents the smaller outlet port of the Venturi chamber [40]. **a**-2 Silicone models of the Ahmed® Glaucoma Valve: (2i) Ahmed^®^ Glaucoma Valve Model FP7; (2ii) Ahmed^®^ Glaucoma Valve Model FX1; (2iii) Ahmed^®^ Glaucoma Valve Model FP8; and (2iv) Ahmed^®^ Glaucoma Valve Model PC7—Ahmed^®^ FP7 with Pars Plana Clip [41]. **b** The Ahmed^®^ ClearPath Glaucoma Drainage Device: (i) model CP350; and (ii) model CP250 [60]. “SA” stands for surface area of the end plate.

A detailed description of each commercially available aqueous shunt, including the Molteno^®^, Baerveldt^®^, and PAUL^®^ implants, Ahmed^®^ Glaucoma Valve, and Ahmed^®^ ClearPath will be given below. These implants are shown in Fig. 2 [25, 38, 39] and Fig. 3 [40, 41].

### Molteno^®^ Glaucoma Drainage Device

The original Molteno implant (Molteno Ophthalmic Limited, Dunedin, New Zealand), shown in Fig. 2a-i, consists of a long silicone tube (inner diameter, ID = 0.34 mm; outer diameter, OD = 0.64 mm) connected to a large 133 mm^2^ polypropylene end plate [42–44]. A double plate version is also available, as demonstrated in Fig. 2a-ii, which allows for a greater IOP reduction due to the increased available space for aqueous absorption in the subconjunctival/sub-Tenon’s space [1, 43, 45, 46]. Although acceptable long-term outcomes were obtained with these early devices, severe postoperative hypotony and hypotony-related complications were often reported as a result of overfiltration [44, 47]. Thus, techniques to address this problem were soon explored, including ligating the tube externally with an absorbable ligature (which degrades after ~6 weeks). This enables the formation of a tissue-capsule over the plate, which then offers some resistance to aqueous humour outflow [1, 25, 48]. Later on, Molteno additionally introduced the Molteno implant with a pressure ridge (see Fig. 2a-iii, iv), designed to further reduce the risk of postoperative hypotony [45]. In this device, the top portion of the (main) plate is divided into two separate chambers, with the help of a thin V-shaped ring, which limits the initial available area for drainage of fluid [43]. The smaller V-chamber, when covered by Tenon’s capsule, serves as a pressure-sensitive valve that regulates the fluid flow into the bleb cavity [1, 45]. Aqueous humour in the V-chamber must therefore overcome the resistance imposed by the tension of the overlying Tenon’s capsule to flow further, which presumably delays fluid drainage thereby preventing severe postoperative hypotony [43]. A new larger single plate Molteno implant with pressure ridge, called Molteno3^®^ S-series, is nowadays preferably used over previous devices. It has a thinner and more flexible episcleral plate which is available in two sizes: 185 and 245 mm^2^, represented in Fig. 2a-v [48, 49]. Other variations of the Molteno implant include a paediatric/microphthalmic implant shown in Fig. 2a-vi, which is a mini version of the original single plate implant designed to fit a microphthalmic globe (abnormally small eye) [45].

### Baerveldt^®^ Glaucoma Implant

The Baerveldt implant (Johnson & Johnson Vision, California, USA) contains a single plate with a larger surface area than any Molteno device [50]. Baerveldt designed this implant in an attempt to provide an easy placement of a large end plate, that should offer greater long-term IOP control, in a single quadrant of the eye. This is not possible with the double plate Molteno devices that require two-quadrant dissection. The Baerveldt implant is comprised of a soft silicone tube (ID = 0.305 mm; OD = 0.635 mm) connected to a soft, pliable, barium-impregnated silicone end plate [43]. The end plate is available in two sizes: 350 mm^2^ that is usually sufficient to manage adult glaucoma (see Fig. 2b-i), and 250 mm^2^ used for individuals with small eyes or when the larger plate cannot be placed (see Fig. 2b-ii) [25]. The plate is additionally equipped with small fenestrations, allowing the growth of fibrous bands through the plate thereby riveting the bleb to the sclera and thus reducing bleb height [43]. The implantation procedure is similar to the Molteno implant, with both devices requiring special techniques to temporarily obstruct flow in the early postoperative period [25]. Despite the use of flow restricting techniques, severe hypotony is still frequently associated with the Baerveldt implant [43].

More recently, another version of the Baerveldt implant was introduced: the Hoffman-elbowed pars plana Baerveldt implant, shown in Fig. 2b-iii. This implant was designed to be inserted into the vitreous cavity, with the distal end of the tube specially modified with an additional small silicone plate (Hoffman elbow) for this purpose [51]. Tube insertion through the pars plana (the posterior part of the ciliary body, represented in Fig. 1a) is indicated in pseudophakic eyes with prior pars plana vitrectomy (procedure where vitreous humour is removed), patients with very shallow anterior chambers, or in patients that underwent corneal transplantation [25, 43, 52].

### PAUL^®^ Glaucoma Implant

The PAUL Glaucoma Implant (Advanced Ophthalmic Innovations, Singapore, Republic of Singapore) is a novel shunt manufactured from medical-grade silicone that differentiates from other aqueous shunts by its smaller lumen diameter (ID = 0.127 mm; OD = 0.467 mm). This device is also comprised of a large surface area end plate for aqueous absorption (342 mm^2^), as shown in Fig. 2c. A recent 12-month follow-up study revealed that the PAUL implant has comparable efficacy with other currently available implants, with almost three quarters of the patients enroled in the study achieving complete surgical success after 1 year. The most significant postoperative complications included shallow anterior chamber, tube occlusion and exposure, hypotony requiring intervention and endophthalmitis-purulent inflammation (inflammation of the intraocular fluids usually due to infection) [38]. As it is a relatively recent implant, more studies are necessary to confirm its long-term efficacy in reducing IOP and its safety profile.

### Ahmed^®^ Glaucoma Valve

The Ahmed implant (New World Medical, Inc., California, USA) is comprised of three parts, represented in Fig. 3a-1: an oblong-shaped end plate, a drainage tube (ID = 0.30 mm; OD = 0.63 mm) and a valve mechanism [40, 49]. The restricting valve is located on the end plate and is comprised of two opposed deformable silicone elastomer membranes pinned together along their edges [53]. These membranes are pretensioned to open at an IOP threshold of 8 mmHg, and to remain closed below this value to reduce risk of hypotony [40]. They create a Venturi-shaped chamber where the inlet cross-section is wider than the outlet, which generates a pressure differential across the chamber. As demonstrated by Bernoulli’s principle [54], the velocity of aqueous entering the larger port of the Venturi chamber (Section A in Fig. 3a-1) increases significantly toward the smaller outlet port (Section B in Fig. 3a-1). This increased exit velocity facilitates the evacuation of aqueous humour from the valve [40]. Although this outflow restriction mechanism embedded in the Ahmed valve appears to decrease to some extent the risk of postoperative hypotony, this is still a very serious complication that affects a significant proportion of patients. This might be associated with valve malfunctioning, as in vitro studies have shown a high variability of the opening and closing pressures [55, 56].

Different models of the Ahmed valve are available, varying in size, shape, and number of end plates [47]. All tubes are made of silicone, and the end plates are made of polypropylene or silicone. Figure 3a-2 shows the silicone models, which have been shown to offer improved IOP reduction, as well as a lower incidence of excessive encapsulation when compared to the polypropylene models [57, 58]. A newer design of the Ahmed valve made of porous high-density polyethylene polymer is also available, whose pores are believed to allow for tissue integration and vascular ingrowth resulting in thinner and more vascular bleb capsules. Studies comparing this concept with the prior silicone models did not find significant differences in final IOP outcomes, although less “hypertensive spikes”, which usually occur several months after surgery, were observed with the newer porous polyethylene Ahmed valve [59].

### Ahmed^®^ ClearPath glaucoma drainage device

New World Medical has recently launched a new valveless glaucoma drainage device: the Ahmed® ClearPath. This implant consists of a medical-grade silicone tube (ID = 0.305 mm; OD = 0.635 mm) secured to a flexible, barium-impregnated silicone episcleral plate that conforms to the natural shape of the globe. Two models CP250 and CP350 are available, shown in Fig. 3b, covering surface areas of ~250 and 350 mm^2^. The CP350 model is positioned more posteriorly to avoid muscle attachment points, while the CP250 model is a single quadrant implant that fits between the muscles. Suture fixation points are positioned more anteriorly on the ClearPath than on other valveless drainage devices, making it easier to secure the implant to the eye. The device is supplied with a polypropylene ripcord (preloaded in the lumen of the tube) to prevent early hypotony, and a 23-gauge needle. The Ahmed ClearPath received clearance from FDA in 2019 via the 510(k) pathway with the Baerveldt Glaucoma Implant as predicate device. When comparing both devices in terms of pressure/flow properties and effectiveness of tube occlusion utilising a ripcord, the results establish that the Ahmed ClearPath and Baerveldt implant are equivalent [60, 61].

### Trabeculectomy-modifying device—EX-PRESS^®^

The EX-PRESS Glaucoma Filtration Device (Alcon Laboratories, Inc., Texas, USA) is a miniature, tube-like implant made of medical-grade stainless steel (316LVM) that was designed with the intention of offering a simple and safe alternative to the classic trabeculectomy [62, 63]. Overall, glaucoma surgery with the EX-PRESS device achieves IOP reduction similar to that of trabeculectomy, but the EX-PRESS procedure is more predictable with less variance of IOP during the early postoperative period [21, 63]. However, complications such as erosion, displacement, and blockage of the implant, as well as hypotony-related complications are commonly reported [62, 63]. For this reason, and due to the high cost of the device itself, trabeculectomy might still be preferred over this implant [64].

### Minimally invasive glaucoma surgery (MIGS) devices

Although aqueous shunts have been proven to be effective at lowering IOP in glaucoma patients and in preventing disease progression, they have a long list of potential complications [22, 27, 65–67]. Hence, there is a clinical need for better designed devices which must have equivalent IOP-lowering capabilities as compared to traditional incisional surgeries but with an improved safety profile. To meet this clinical need, a number of procedures and devices have recently been developed labelled as either minimally invasive or MIGS [2, 68, 69]. For the current purpose, we will limit the discussion to implantable devices. The criteria for meeting the definition of a MIGS device are somewhat controversial. On the one hand, FDA defines a MIGS device as “a type of IOP-lowering device used to lower IOP using an outflow mechanism with either an ab interno or ab externo approach, associated with little or no scleral dissection and minimal or no conjunctival manipulation” [70]. On the other hand, the European Glaucoma Society Guidelines state that “only the ab interno non-bleb-forming procedures can be defined as MIGS” [71]. The ab interno approach targets the trabecular meshwork or suprachoroidal space from within the anterior chamber, whereas in an ab externo procedure the trabecular meshwork is reached or a device is implanted into the anterior chamber from the outside of the eye, after a subconjunctival/sub-Tenon’s or scleral flap is created [69]. In this review, we will follow the current FDA definition of a MIGS device, because in our opinion, irrespective of whether they are implanted through an ab interno or ab externo approach, the most important is the final outcome: IOP reduction with reduced tissue destruction, a relatively high safety profile, short surgery time, simple instrumentation, and rapid recovery [24].

MIGS devices can be classified into three main categories based on the site of anatomical intervention and augmentation: (1) Schlemm’s canal MIGS devices, where trabecular outflow is increased by bypassing the trabecular meshwork and directing aqueous humour into Schlemm’s canal; (2) suprachoroidal MIGS devices, where uveoscleral outflow is increased via implantation of suprachoroidal shunts; and (3) subconjunctival MIGS devices, where a drainage pathway is created into the sub-Tenon’s space [23, 72].

### Schlemm’s canal MIGS devices

**Fig. 4:**
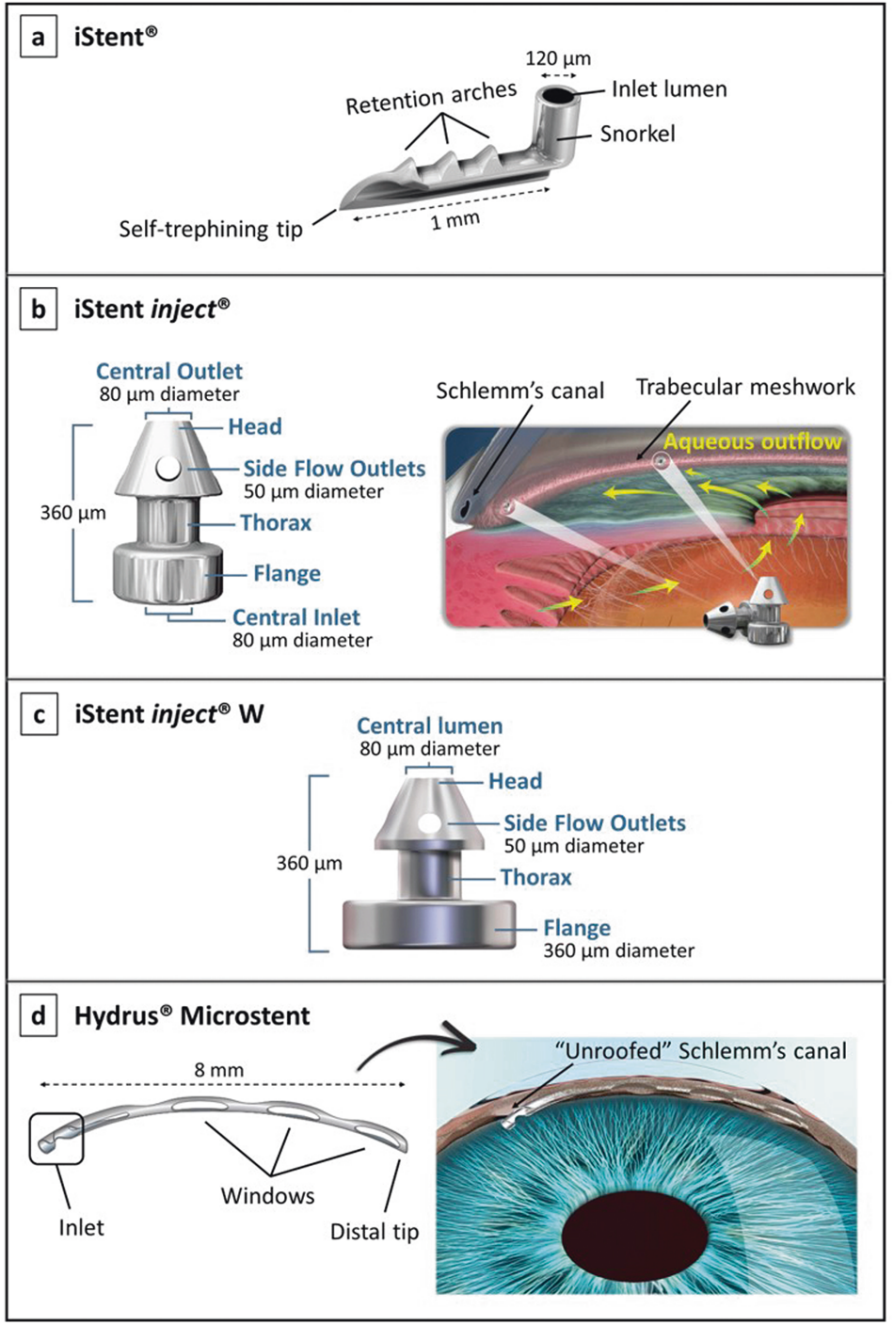
Schlemm’s canal MIGS devices. **a** The first-generation iStent^®^, showing its self-trephining tip that is inserted into Schlemm’s canal via a sideways sliding technique, its retention arches which help maintaining the device in position, and its lumen that faces the anterior chamber [68]; image courtesy of Glaukos Corporation. **b** The second-generation iStent inject^®^, showing its head containing four side ports and designed to fit into Schlemm’s canal, and its flange with an inlet lumen that faces the anterior chamber as illustrated in the figure on the right side [68, 74]; images courtesy of Glaukos Corporation. **c** The iStent inject^®^ W, showing its larger flange diameter as compared with the previous version iStent inject^®^ [79]; image courtesy of Glaukos Corporation. **d** The Hydrus^®^ Microstent, showing its three open windows along its anterior surface and its placement in the eye (figure on the right) [75, 76]; images courtesy of Ivantis Inc.

Since the trabecular meshwork was originally considered the main site of resistance to aqueous humour outflow, bypassing this structure and directing aqueous flow from the anterior chamber directly into Schlemm’s canal seemed to be a reasonable approach [13, 73]. Currently, there are four Schlemm’s canal MIGS devices available: the iStent^®^, iStent inject^®^, iStent inject^®^ W, and the Hydrus^®^ Microstent [23]. These devices are shown in Fig. 4 [68, 74–76]. They are all inserted via an ab interno approach, under gonioscopic view.

The first-generation iStent (Glaukos Corporation, California, USA), represented in Fig. 4a, is a heparin-coated titanium, “L”-shaped device which, via an ab interno incision and using a preloaded inserter, is placed through the trabecular meshwork into Schlemm’s canal [75]. The canal portion of the iStent, designed to fit into Schlemm’s canal, is an open half-pipe which contains a curved convex side that lies against the inner wall of the canal. Perpendicular to this portion, there is a tubular, small “snorkel” facing the anterior chamber, which serves as a conduit for aqueous to bypass the inner wall of Schlemm’s canal and trabecular meshwork, thus increasing outflow [2, 75, 77]. In general, iStent implantation is associated with a good safety profile, with the most common complication being transient hyphema. Stent malposition and obstruction also occur, which is often solved by laser intervention, or ultimately, by implant removal and replacement [68]. There are no reports yet of serious complications such as choroidal effusion, persistent hypotony, bleb formation, or endophthalmitis [78]. Besides, the placement of more than one iStent in the same eye was proven to have an additive effect in lowering IOP. Hence, a second-generation iStent was developed, called iStent inject (Glaukos Corporation, California, USA), shown in Fig. 4b [24, 72, 77]. The iStent inject is smaller and is a conical-shaped device also made out of heparin-coated titanium [69, 78]. In contrast to the previous iStent, this device is administrated via auto-injection, where up to two devices can be delivered into Schlemm’s canal with a single injector device. This allows the surgeon to inject two iStents while entering the eye only once, thus reducing surgical time and the risk of adverse events [68, 77]. More recently, a new version of the second-generation iStent inject, the iStent inject W, has been developed, featuring a wide flange at its base to optimise stent visualisation and placement. The diameter of the flange was increased from 230 to 360 microns, as can be seen in Fig. 4c. This device received FDA approval in 2020 [79].

The Hydrus Microstent (Ivantis, Inc., California, USA), illustrated in Fig. 4d, is a scaffold-like implant inserted ab interno into the Schlemm’s canal to maintain the canal open, thus enhancing trabecular outflow [80]. It is flexible in nature and is comprised of nitinol, a biocompatible nickel titanium alloy. It is open posteriorly along its length and has three open windows along its anterior surface. Using this device, Schlemm’s canal can be dilated by up to four to five times the natural cross-section of the canal, and along one fourth of its length thus targeting multiple collector channels. However, implantation of this device is also more difficult than other Schlemm’s canal MIGS devices [2, 75]. The Hydrus implant is reported to be generally safe, and complications are infrequent. As with all other ab interno approaches, the most commonly reported complication is transient hyphema [77, 78]. A study comparing the Hydrus Microstent with two iStent inject implants revealed that, while the IOP results and the safety profile were similar between the two devices, the implantation of the Hydrus Microstent more often reduced the need for postoperative glaucoma medications. However, more studies are necessary to validate these results and further prove the efficacy of these implants [30].

### Suprachoroidal MIGS

**Fig. 5:**
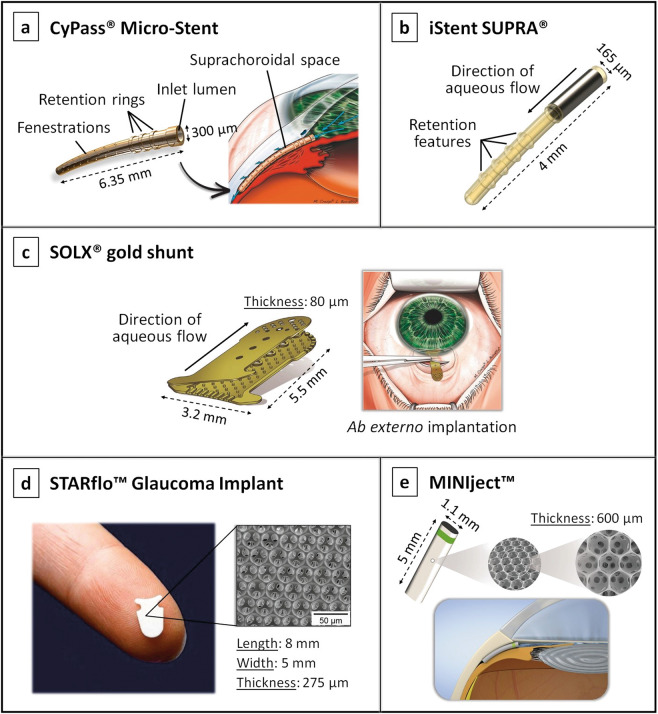
Suprachoroidal MIGS devices. **a** The CyPass^®^ Micro-Stent showing its fenestrations through which aqueous humour flows into the suprachoroidal space, its retention rings which help anchoring the device, and its placement in the eye (figure on the right); image reproduced with permission from [81]. **b** The iStent SUPRA®, with its retention rings; image reproduced with permission from [1]. **c** The SOLX^®^ gold shunt, showing its two gold plates and its implantation procedure performed through an ab externo incision (figure on the right); image reproduced with permission from [81]. **d** The STARflo™ Glaucoma Implant showing its anvil-like head designed to prevent extrusion from the anterior chamber, and its multi-porous geometry characterised by hollow spheres arranged in a regular network pattern [1, 82]; image reproduced with permission from [92]. **e** The MINIject™ device showing its multi-porous structure and its positioning in the eye [35].

In contrast to the Schlemm’s canal-based MIGS that aim to improve the trabecular outflow pathway, suprachoroidal MIGS devices aim to take advantage of the uveoscleral pathway to reduce IOP [72]. These devices, shown in Fig. 5, include the CyPass^®^ Micro-Stent, iStent SUPRA^®^, SOLX^®^ gold shunt, STARflo™ Glaucoma Implant and the MINIject™ [1, 35, 81, 82].

The CyPass Micro-Stent (Alcon Laboratories, Inc., Texas, USA) is a device made of biocompatible polyimide [75]. It is fenestrated along its length as can be seen in Fig. 5a, with pores of 76 µm in diameter which allow for aqueous outflow [2, 68]. The stent is threaded through a guidewire and applicator into the supraciliary space (via an ab interno procedure), where it is then anchored passively with moulded-in retention rings [68, 77]. Even though early clinical studies have shown that implantation of this device leads to slight reduction in IOP and glaucoma medications, in August 2018 the CyPass was withdrawn from the global market due to safety concerns about endothelial cell loss resulting from mispositioned devices [2, 18, 83, 84].

The iStent SUPRA (Glaukos Corporation, California, USA), shown in Fig. 5b, is a different iteration of the two iStent Schlemm’s canal MIGS devices discussed earlier. It is a small heparin-coated device composed of polyethersulfone and titanium, which is slightly curved to follow the curvature of the sclera and has ridges to improve implant retention [2, 72]. Like the CyPass microstent, the iStent SUPRA is inserted through an ab interno incision [68].

The SOLX gold shunt (SOLX, Inc., Massachusetts, USA) is a rectangular-shaped device made of 99.95% pure gold, see Fig. 5c [85]. The device is composed of two gold plates welded together and containing 19 microchannels—initially ten closed and nine open [86, 87]. Holes at both ends of the device allow aqueous humour to flow through the channels from the anterior chamber into the suprachoroidal space [2]. The main novelty associated with this device was that it allows the surgeon to control aqueous humour outflow postoperatively if needed, by using a titanium-sapphire laser to open the channels [75]. Nevertheless, the SOLX microshunt never received FDA approval due to high rates of failure caused by significant fibrotic tissue formation both inside the shunt grid and around the device, which cannot be totally resolved by applying laser shots to increase outflow. Additionally, serious complications following implantation have been reported, such as retinal detachment, endophthalmitis and suprachoroidal haemorrhage [76, 88–91].

The STARflo (iStar Medical, Wavre, Belgium) is shown in Fig. 5d, and is an innovative MIGS device made of a flexible silicone microporous material named “STAR” derived from NuSil med-6215 (a silicone elastomer). Its multi-porous geometry, comprised of a highly organised network of hollow spheres, was designed to promote biointegration from the surrounding tissues into the material, thereby maintaining the drainage efficiency on a long-term [92]. The device is composed of an anvil-like head designed to prevent extrusion from the anterior chamber, and a body that is positioned into the supraciliary space through an ab externo approach [86]. As the implant is relatively new, few clinical trials exist attesting its efficacy and safety [87]. However, a recent 24-month follow-up study revealed that the implant had failed to provide a safe and effective long-term alternative to conventional glaucoma surgeries, with unsatisfactory reduction in IOP [34]. Postoperative complications, such as corneal decompensation, hypotony, choroidal haemorrhage, and unspecified macular changes have also been reported [34, 86].

The MINIject (iStar Medical, Wavre, Belgium) is another suprachoroidal MIGS device composed of the same STAR material and porous structure as the STARflo device, as illustrated in Fig. 5e. It has a green ring on its surface which is used to confirm adequate implantation. The results obtained from the first human trial indicated that the MINIject was able to reduce IOP in patients with mild-to-moderate glaucoma, and to maintain a stable IOP control without topical medication. No serious adverse events were reported, however, further studies are required to prove the long-term safety of this new device [35].

In general, although the suprachoroidal pathway is an interesting variant of MIGS devices, the results are not very successful yet due to a high risk of fibrosis and/or possibly severe complications.

### Subconjunctival MIGS

**Fig. 6:**
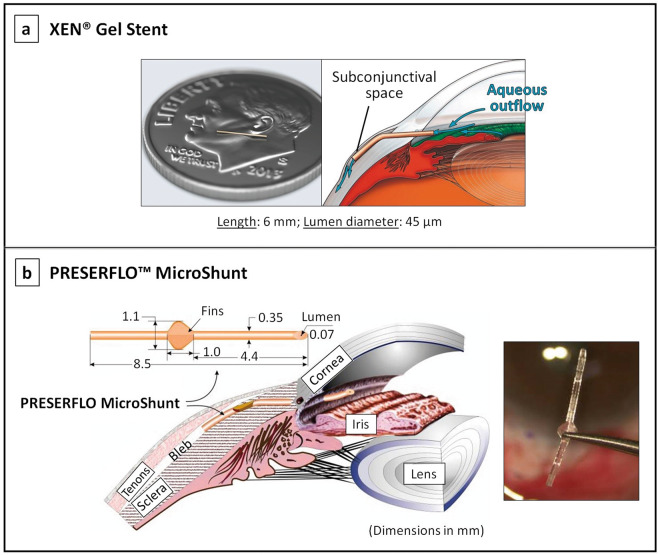
Subconjunctival MIGS devices. **a** The XEN^®^ Gel Stent showing its small dimensions and its positioning in the subconjunctival space [81, 93]; image reproduced with permission from [81]. **b** The PRESERFLO™ MicroShunt showing its dimensions (mm) and placement in the eye [94].

Contrarily to the MIGS strategies described above, the subconjunctival route is fundamentally non-physiological as aqueous humour does not naturally flow into the subconjunctival/sub-Tenon’s space [72]. There are currently two subconjunctival MIGS devices, which are shown in Fig. 6: the XEN^®^ Gel Stent and the PRESERFLO™ MicroShunt [81, 93, 94]. The implantation of both devices is augmented with intraoperative application or injection of mitomycin C (MMC, antifibrotic agent) to reduce the risk of subconjunctival fibrosis.

The XEN gel stent (Allergan, Inc., Dublin, Ireland) targets the subconjunctival space for aqueous drainage via an ab interno approach, see Fig. 6a [18]. The device is a hydrophilic tube made of porcine gelatin cross-linked with glutaraldehyde [2, 18]. During the implantation procedure, the XEN implant hydrates and swells in place to become a soft non-migrating drainage channel that is tissue-conforming [75]. The available evidence suggests that there is a reduction in IOP as well as in the number of postoperative glaucoma medications required, which presents a relatively good safety profile [95, 96]. However, a high need for postoperative bleb intervention (needling) after the implantation of this device is commonly reported among studies [97].

The PRESERFLO MicroShunt (Santen, Osaka, Japan), formerly known as the InnFocus MicroShunt, is flexible tube made from a highly biocompatible, bioinert material called poly(styrene-*block*-isobutylene-*block*-styrene), or SIBS (see Fig. 6b) [94]. Located halfway down the microshunt is a 1.1 mm wingspan fin that sits within a shallow pocket in the sclera, which prevents migration of the device into the anterior chamber and also helps minimise aqueous leakage around the tube [75, 94, 98]. The SIBS material from which this implant is made is biostable and its inert nature evokes minimal inflammation and scar tissue formation. Initial studies in rabbit eyes comparing the tissue response to SIBS versus silicone tubes indicated that the silicone rubber stimulates inflammation and promotes development of a fibrotic capsule around the device that quickly becomes non-functional, while the SIBS tubes demonstrated minimal encapsulation with continuous aqueous outflow after 1 year [75, 99]. Subconjunctival inflammation induced by silicone has been reported in other studies [100]. Results from a recently completed clinical trial assessing the safety and effectiveness of the PRESERFLO MicroShunt indicate that the device is able to significantly reduce IOP in patients with mild-to-severe glaucoma, and to maintain healthy IOP levels in the long-term [28, 101]. Complications associated with this device are generally transient and self-limiting, and include early hypotony, shallow anterior chamber, choroidal effusion and hyphema. No cases of infections, migrations, erosions, or other serious bleb-related complications have been reported to date [28, 94, 102, 103].

### Comparison between glaucoma implants

When comparing the different glaucoma implants, the most important factors to consider include short- and long-term IOP control, adjunctive use of glaucoma medications, and postoperative complications [104]. The degree of IOP reduction is a surrogate for successful glaucoma therapy, as IOP is the only known manageable risk factor for glaucoma progression. As such, it serves as an important measure of surgical success and is a good indicator of the effectiveness of a glaucoma drainage device [66].

Recent randomised clinical trials have compared the efficacy and safety of the three conventional glaucoma implants: Molteno, Baerveldt, and Ahmed implants. The Ahmed Baerveldt Comparison (ABC) and Ahmed Versus Baerveldt (AVB) studies are two relevant multicenter, randomised trials comparing the most frequently used aqueous shunts: the Ahmed FP7 valve (see Fig. 3a-2i) and the Baerveldt 101–350 implant (see Fig. 2b-i) [27, 66, 100]. The effectiveness in reducing IOP reported for both devices in the AVB study is shown in Table 1. Overall, the success rate of IOP control was found to be very similar between these devices, with long-term percentage of reduction in IOP around 50% from the preoperative value. The Baerveldt implant produced slightly greater IOP reduction with fewer adjunctive medications as compared with the Ahmed valve during 5 years of follow-up, which can be explained by the larger end plate of the 350 mm^2^ Baerveldt implant: larger surface area plates are associated with greater IOP reduction [20]. On the other hand, due to the built-in flow restriction valve of the Ahmed implant, complications associated with overfiltration and subsequent hypotony in the immediate postoperative period appear to occur less frequently [47]. However, ultimately most failures of glaucoma implants are the result of high IOP as opposed to low IOP. The Ahmed valve also showed greater IOP reduction in the early postoperative period as compared with the Baerveldt implant, although this is expected as the Baerveldt tube is occluded with a temporary suture during the first few weeks after surgery to prevent early hypotony. The most common postoperative complication was bleb encapsulation resulting in elevated IOP, although it was more frequently associated with the Ahmed valve. This may be explained by the early exposure of the Ahmed bleb to the mechanical stresses imposed by the aqueous outflow, as well as exposure to proinflammatory factors incited by surgery, which may produce more vigorous scarring of the fibrous capsule surrounding the end plate [27, 66]. This bleb encapsulation might additionally explain the lower IOP reduction achieved with the Ahmed valve in the long-term. In contrast, delaying flow may elicit less fibrous reaction, which potentially explains the lower incidence of bleb encapsulation with the Baerveldt implant [26, 27, 66, 100, 105].

The Ahmed valve was also compared to the single plate Molteno implant in a prospective randomised study, in which results are very similar to those reported in the AVB and ABC studies [26]. After 2-year follow-up, the Molteno implant showed significantly lower IOPs compared to the Ahmed valve, although it was associated with higher IOPs and mean number of antiglaucoma medications within the first postoperative month. On the other hand, the Ahmed valve was associated with higher rates of bleb encapsulation [26]. In summary, these findings suggest that the Molteno or Baerveldt implants may be a better choice for patients with a low long-term IOP target. However, patients need to be followed closely in the early postoperative period while the tube is ligated in the event a sudden increase in IOP occurs. The Ahmed implant may especially be an appropriate option for patients who need immediate postoperative IOP reduction and have moderate long-term IOP targets. Currently, the Ahmed and the Baerveldt implants are the most commonly used plated glaucoma shunts worldwide [26, 27, 66].

While conventional glaucoma implants are generally preferred for patients with more severe glaucoma, MIGS devices are currently considered when: (1) IOP reduction goals are more modest; (2) the glaucoma disease is newly diagnosed; and/or (3) the optic nerve damage is only mild to moderate [106]. The reason behind this is that IOP reduction tends to be less pronounced with the majority of MIGS devices as compared with more conventional implants and the trabeculectomy-modifying EX-PRESS device, as can be inferred from Table 1 [2]. A possible exception to this is the PRESERFLO MicroShunt, a subconjunctival MIGS device that seems to have the potential to be as effective as conventional implants in reducing IOP [28, 107]. However, this efficacy was found to be dependent on the concentration of MMC exposure during implantation. Two-year results from an international multicenter prospective trial presented at the World Glaucoma Congress revealed better IOP and medication outcomes in patients treated with 0.4 mg/ml MMC as compared to patients treated with 0.2 mg/ml MMC [103]. Nevertheless, even when lower concentrations of MMC are used, the PRESERFLO MicroShunt appears to perform better than other MIGS devices. In a recent study comparing the XEN Gel Stent and the PRESERFLO MicroShunt where the same concentration of MMC was applied (0.2 mg/ml), it was reported a reduction of IOP of 28.1% and 39.8% at 2 years of follow-up for both devices, respectively [108]. This may indicate that the PRESERFLO MicroShunt is more effective in reducing IOP as compared with the XEN device. This finding may be associated with the high rate of bleb encapsulation that is frequently reported with the latter device [108, 109]. The lower rate of bleb encapsulation with the PRESERFLO MicroShunt may be due to the biocompatibility of the SIBS material, which was designed specifically to be non-degradable, ultra-pure and therefore non-inflammatory thereby generating less tissue fibrosis [110]. Nonetheless, more robust data from long-term clinical trials is required to determine the relative efficacy and safety of these devices.

Although possibly more effective at lowering IOP, the subconjunctival MIGS devices, as bleb-forming procedures, carry risks of bleb-related complications. Regardless of their small luminal diameter, which provides increased resistance to prevent overfiltration, some cases of early hypotony have still been reported. In Schlemm’s canal MIGS devices the risk of hypotony is significantly reduced, as postoperative IOP cannot fall below the episcleral venous pressure. This represents the main advantage of Schlemm’s canal MIGS devices [111]. However, for the same reason, the Schlemm’s canal devices should be avoided in glaucomatous eyes with raised episcleral venous pressure, as they yield disappointing outcomes in terms of IOP reduction [112]. Additionally, in case the IOP decreases below episcleral venous pressure, there is a high risk of blood reflux into the anterior chamber, causing hyphema, which represents the most common postoperative complication following Schlemm’s canal procedures [106, 111]. Another important limitation of both Schlemm’s canal and especially suprachoroidal MIGS devices is the fact that excessive wound healing can occur in the region of implantation, which may (and frequently) leads to device obstruction [76]. This results in increased IOP and potential need for additional interventions. One important reason behind the high rate of failure resultant from excessive fibrosis in these devices is that there is currently no approach to apply antifibrotic agents safely to the site of implantation without risk of intraocular toxicity [2, 113]. Device obstruction is an important limitation among all MIGS devices, most importantly due to their small lumen diameter. Despite being advantageous in decreasing the risk of hypotony, smaller lumens are at risk of blockage by fibrin, iris pigment, blood, vitreous, and/or lens fragments.

With regard to the current state of MIGS, limited data about the long-term efficacy and safety of these procedures are available until now. Additionally, lack of study standardisation, randomised controlled trials, and incomplete knowledge of ideal patient selection make it problematic to reach robust conclusions. Most evidence is derived from non-comparative studies and before-after studies. Furthermore, concomitant application of different therapies in clinical studies with MIGS implants, such as combination with cataract surgery, makes it difficult to do a proper evaluation and comparison of the results obtained. Thus, a standardisation of future studies is urgently needed [111]. In March 2009, the World Glaucoma Association (WGA) has published guidelines for conducting clinical trials with recommendations regarding methodology, definition of success, ethical considerations, reporting of postoperative complications, economic evaluation, and statistical analysis. However, a study from Mathew et al. determining the extent of adherence of MIGS trials to the WGA guidelines concluded that, from the studies evaluated, there was poor adherence (45.6 %) to the WGA guidelines [114, 115]. There is additionally still limited evidence on the cost-effectiveness of MIGS. The downside of many of the MIGS devices is their high cost in comparison to both trabeculectomy and traditional devices—the cost of the MIGS is typically a factor of two higher than that of traditional devices (in the Netherlands ~€ 1200 versus € 650). It remains unclear whether the cost of using MIGS is outweighed by cost savings through decreased medication and reduced need for further interventions [116]. Recently published literature assessing the economic outcomes of MIGS devices/procedures concludes that most of the economic studies available so far do not consider indirect costs, costs related to postoperative complications and follow-up, and quality of life. These gross-costing studies use averages and assumptions, thereby decreasing the transparency and ability to deliver consistent estimates. Hence, future economic analyses of MIGS devices should be conducted through micro-costing studies, which include every input consumed in a patient’s management. These studies will increase the precision and transparency in estimating costs and better reflect the use of resources. Another limitation of current economic evidence on MIGS, which is shared by most economic analyses, is that the reported findings may not be generalised between countries since the healthcare system and costs are different [115, 116]. To conclude, new and better designed cost-effectiveness studies are warranted to gain the MIGS devices a place within the total treatment armamentarium for glaucoma.

### Future directions

For patients with mild to moderate glaucoma, Schlemm’s canal or suprachoroidal MIGS devices are a promising treatment option. Since these procedures do not involve the formation of a filtering bleb, they avoid the bleb-related complications that the subconjunctival devices are susceptible to. Additionally, they preserve the conjunctiva in the event future incisional surgeries are required [87]. However, the longevity and success of these devices depend on the absence of excessive fibroblastic proliferation and scarring both within the devices or around them. Thus, the development of new methods of application of antifibrotic agents for these devices seems appropriate, especially for suprachoroidal devices. Alternatively, preventing excessive fibrosis may be achieved by using optimal biocompatible materials that induce minimal tissue reaction [2].

The ideal MIGS device for more severe cases of glaucoma would produce an IOP-lowering effect similar to trabeculectomy and conventional drainage devices, but with an improved safety profile. The newer subconjunctival, bleb-forming devices appear to be closer than other MIGS devices in achieving this goal. However, although the rate of hypotony and bleb-related complications seems to be lower with these devices as compared with more traditional surgeries, their occurrence is still significant [117].

**Fig. 7:**
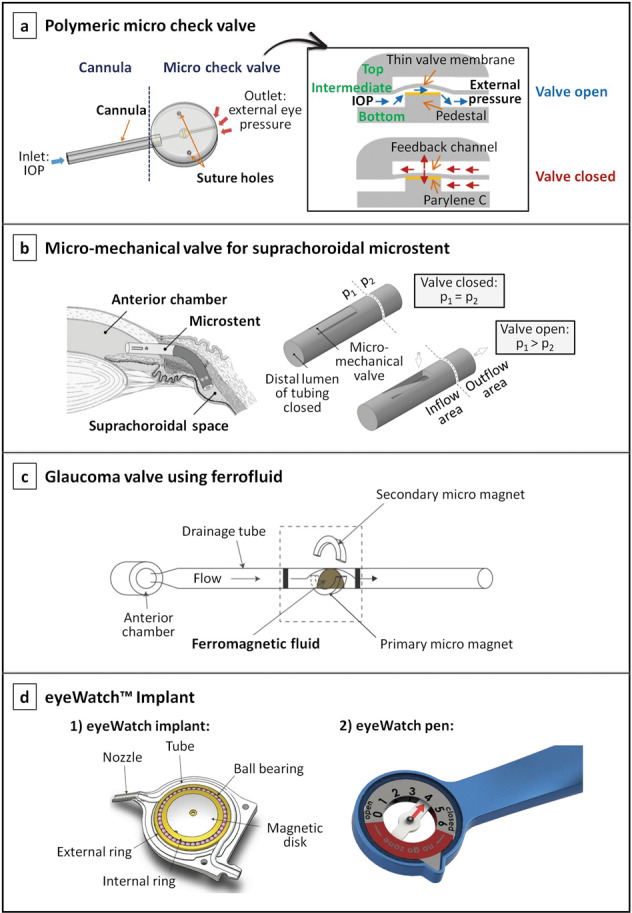
Proof-of-concept of innovative passive/active valve mechanisms for glaucoma drainage devices. **a** Illustration of a glaucoma drainage device consisting of a cannula (drainage tube) and a micro check valve; the cross-sectional view of the valve and working principle are represented on the right; image reproduced with permission from [119]. **b** Concept of a microstent for drainage of aqueous humour into the suprachoroidal space, showing its flap-like micro-mechanical valve that opens when the pressure in the inflow area (p_1_) is higher than the pressure in the outflow area (p_2_); image reproduced with permission from [120]. **c** Representation of a ferrofluidic valve architecture for a glaucoma drainage device [121]. **d** The eyeWatch system, which is comprised of: (1) the eyeWatch implant, depicting details of its valve mechanism [122]; and (2) the eyeWatch pen, which is the control unit of the eyeWatch system [123]; images courtesy of Rheon Medical SA.

To minimise the incidence of hypotony, valves have been incorporated in long-tube glaucoma implants, e.g. the Ahmed valve, in an attempt to increase the flow resistance and to provide better IOP control. Even though the Ahmed valve is associated with low rates of early postoperative hypotony, evidence suggests that hypotony continues to occur [118]. To overcome this, other innovative concepts of passive valves, as well as active valves, have been proposed. In Fig. 7 some of these proof-of-concept valve mechanisms are represented [119–123].

A number of passive flow-control mechanisms based on flaps, membranes, or ferromagnetic substances have been described in the literature [119–121, 124, 125]. Park et al. proposed a novel polymeric micro-check valve for a glaucoma drainage device, which is comprised of three layers as shown in Fig. 7a [119]. The intermediate layer is composed of a thin valve membrane resting on a pedestal, designed to lift upwards when the IOP is greater than the sum of the cracking pressure (the minimum upstream pressure required to open the valve) and external pressure on the outlet side. When the valve opens, a space is created between the valve and the pedestal, allowing the aqueous humour to flow further. Conversely, when the IOP is less than the sum of these pressures, the valve membrane returns to its original closed position, thereby avoiding postoperative hypotony. In this work, the pedestal was specially elevated by coating it with Parylene C, in order to induce a prestress in the valve membrane that allows for a precise opening pressure to be achieved (around 10 mmHg). Another micro-mechanical valve embodiment designed for a suprachoroidal implant was proposed by Siewert et al. [120]. The valve, represented in Fig. 7b, exhibits a tongue-like shape and is located in the inflow area (anterior chamber), positioned in the wall of the drainage tube. The authors claimed that previous micro-check valves with direct contact between the valve membrane and the valve seat (pedestal) present high risk of stiction, and thus failure in IOP control, especially in a long-term application. Hence, they proposed this flap-like valve mechanism where no directly contacting components exist. Paschalis et al. proposed a quite different and innovative concept for a passive glaucoma valve, based on ferromagnetic nanoparticles, see Fig. 7c [121]. A ferrofluid was used for the design of the valve, consisting of water-immiscible ferromagnetic nanoparticles that were dispersed in a fluorinated oil as a carrier liquid. Two permanent magnets were also part of the valve system: one placed next to the tube sub-section containing the ferrofluid droplet to hold it from moving with the flow, and the other was placed in the opposite side to adjust the pressure required to bend the droplet and initiate flow. In vitro tests proved that the ferromagnetic valve provided flow occlusion at a pressure of 7 mmHg and flow initiation at a pressure of 10 mmHg [121].

The main advantage of passive valves is that they are power-free, simple to operate, and generally easier to fabricate as compared to active valves. However, active valves allow for the ophthalmologist to precisely and actively adjust the resistance to the aqueous humour outflow to achieve the desired IOP. This allows for a non-invasive, patient-specific IOP management. An example of a device incorporating an active valve is the eyeWatch™ Implant (Rheon Medical SA, Lausanne, Switzerland), the world’s first commercially available adjustable glaucoma implant that received CE mark in 2019. The eyeWatch system features the eyeWatch implant, acting as an adjustable faucet, and the eyeWatch Pen, used to tune the flow resistance of the implant by inducing variable compression of the drainage tube, see Fig. 7d [122, 126]. This compression is achieved by rotating a magnetic disk present inside the implant, which enables the fluidic resistance to be adjusted in order to maintain the IOP within the optimal clinical-targeted range. This is possible by using the eyeWatch Pen, the external control unit containing a compass in one side, which measures the magnetic disk position, and a magnet in the other side, which adjusts the compression of the tube. A study comparing the efficacy and safety of the eyeWatch connected to a Baerveldt implant versus the Ahmed valve reported no cases of hypotony in the eyeWatch group as compared with the 33% of the patients implanted with the Ahmed valve where hypotony-related complications were observed [127]. Furthermore, initial clinical results with the eyeWatch suggests that it prevents IOP spikes from occurring by fine-tuning the flow resistance of the device when required, thus promoting smooth pressure transitions that may mitigate the tissue response. Additionally, five patients with the eyeWatch implanted underwent an MRI for nonophthalmic reasons, and no cases of discomfort during imaging were reported. Moreover, the imaging artifacts created by the implant were not clinically significant. Patients did, however, require adjustment of the magnetic disk back to its previous position set before the MRI. Nonetheless, as this device is relatively new, further studies are necessary to prove its long-term efficacy and MRI compatibility [128].

Concepts of temporary valves have also been described. Siewert et al. developed a biodegradable flow resisting polymer membrane designed to fit the inlet area of a glaucoma microstent [129]. The authors claimed that the biodegradable membrane would allow for controlled drainage in the early postoperative period and maximised flow capacity at 6 months when degradation is complete. Olson et al. proposed a similar flow restricting mechanism, using a semi-permeable membrane positioned at the tip of a drainage tube that can be ruptured with laser non-invasively after surgery [130]. Initially, the intact membrane will provide high resistance to aqueous humour outflow, to minimise hypotony. Then, when the ophthalmologist determines that the conjunctival wound is stable, the anterior surface of the membrane can be perforated using laser shots to increase fluid flow [130, 131]. The main disadvantage of these concepts is that flow control is only possible during a short-term period (i.e. temporarily).

To help improve the tissue response to MIGS devices implanted subconjunctivally, local drug delivery systems have also been developed. Antimetabolites such as MMC and 5-fluorouracil have been administrated to the subconjunctival space before and during surgery to delay the fibrotic response and improve long-term success [132]. However, potential complications exist with over-administration of these drugs, such as blebitis/bleb-related infection, endophthalmitis, bleb leakage, and conjunctival erosion. The incidence of these complications may be reduced with a sustained slow release of antimetabolites to the site of implantation. This can be achieved, for example, by impregnating the antimetabolite into a biodegradable film, which is then placed on the subconjunctival space at the time of device implantation. The biodegradable film will release the antimetabolite in a controlled manner during the postoperative period, which may benefit the tissue response [133]. Another factor influencing the tissue reaction is the surface topography of the implant, as it constitutes the major site of interaction with the surrounding tissue. Thus, a proper adjustment of the topographic features as well as surface chemistry of the implant may additionally benefit the wound healing process [134].

### Outlook

Glaucoma remains a leading cause of irreversible blindness in the world, and currently the only proven method to prevent disease progression is lowering IOP. For a large population of glaucoma patients, conventional treatments with pharmacological medication, laser treatment, and surgery are not sufficiently effective and safe, and therefore we have witnessed over the last decades an acceleration in the variety of glaucoma drainage devices as alternative treatment approaches. In this review we have described and evaluated these devices, including conventional aqueous shunts, the trabeculectomy-modifying EX-PRESS^®^ device, and the most recent MIGS devices. The ideal device to be used in more severe cases of glaucoma would be a MIGS device that produces an IOP-lowering effect similar to traditional incisional surgeries, such as trabeculectomy and conventional drainage devices, but with an improved safety profile. The newer subconjunctival, bleb-forming devices currently appear to be the best option in achieving this goal. However, longer term studies of these devices need to be performed, to confirm their efficacy in reducing IOP as compared to that of traditional incisional surgeries. For some patients, such as those with normal pressure glaucoma or very advanced glaucoma that need very low pressures (IOP of 6–10 mmHg), these subconjunctival devices, or any other MIGS devices, may not be sufficient.

Toward the future, reducing the rate of postoperative complications and enhancing the safety profile of current subconjunctival MIGS devices, while maintaining their IOP-lowering efficacy, may be achieved by: (1) integrating an active and non-invasive flow-control mechanism, which should allow for a very precise tuning of the IOP, adapted according to each patient’s need—in particular to help avoid hypotony; (2) using drug delivery systems that release antifibrotic agents in a controlled manner, so that their effect on the implanted site is prolonged and beneficial for the tissue response; and (3) optimising the topography of the implant surface to modulate the fibroblast adhesion.
